# Safety assessment of *Plukenetia volubilis* (Inca peanut) seeds, leaves, and their products

**DOI:** 10.1002/fsn3.633

**Published:** 2018-04-02

**Authors:** Warangkana Srichamnong, Pisamai Ting, Pornsiri Pitchakarn, Onanong Nuchuchua, Piya Temviriyanukul

**Affiliations:** ^1^ Institute of Nutrition Mahidol University Salaya Nakhon Pathom Thailand; ^2^ Department of Biochemistry Faculty of Medicine Chiang Mai University Meung Chiang Mai Thailand; ^3^ Nano Agro and Food Innovation Laboratory National Nanotechnology Center (NANOTEC) National Science and Technology Development Agency Klong Luang, Pathum Thani Thailand

**Keywords:** *Drosophila melanogaster*, Inca peanut, phytotoxins, thermal processing

## Abstract

*Plukenetia volubilis* or Inca peanut is a promising plant with high economic value. Its seeds can be pressed for oil production or roasted and served as a snack, while the dried leaves can be used to make a kind of tea. Although the oil from the cold‐pressed seeds has been proven to be safe for human consumption, little information is known about the other parts of the plant regarding safety. Thus, the aim of this study was to investigate the naturally occurring phytotoxins, including saponins, total alkaloids, and lectins in fresh and roasted Inca peanut seeds and leaves. In addition, cytotoxicity on several normal cell types including human peripheral blood mononuclear cells, human embryonic kidney cells, human hepatic stellate cells, and mouse fibroblasts as well as *in vivo* mutagenic properties was studied. This study showed that fresh Inca peanut seeds and leaves contain saponins, alkaloids, and lectins. However, roasting enables the reduction in alkaloids, saponins, and possibly lectins, suggesting that these phytotoxins become unstable under heat. Furthermore, Inca peanut seeds and leaves, especially after roasting, are safe to a variety of normal cell lines and do not induce DNA mutations in *Drosophila* expressing high biotransformation system. In conclusion, the data in this study indicated that high and chronic consumption of fresh seeds and leaves should be avoided. Heat processing should be applied before the consumption of Inca peanut seeds and leaves in order to reduce phytotoxins and potential health risks.

## 1 INTRODUCTION

Alkaloids, lectins, and saponins are the main groups of naturally occurring toxins of plant origin (Phytotoxins), which are secondary metabolites and could be present in many parts of plants, including the seeds, leaves, roots, and bark. Plant secondary metabolites play a vital role as a defending mechanism against predators and pathogens. The consumption of some plants may cause mild to severe toxicity to wildlife, livestock, and humans by either direct toxicity or indirect toxicity (as antinutritional compounds) (Speijers et al., 2010).

Alkaloids are secondary metabolite compounds found in plants, fungi, bacteria, and animals (Dolan, Matulka, & Burdock, 2010). They can be classified into three types on the basis of structure: (i) true alkaloid (heterocyclic with nitrogen inside), (ii) pro‐alkaloid (heterocyclic without nitrogen), and (iii) pseudoalkaloid (produced from amino acid) (Aniszewski, 2007). The functions of alkaloids in plants include defending against consumption by herbivores, acting as a growth hormone, and communication of plant cells (Aniszewski, 2007). Alkaloids can be isolated from all parts of plants. Toxicosis by alkaloids exhibits a wide range of systematic effects, such as gastrointestinal toxicity, kidney toxicity, and genotoxicity. In addition, alkaloids such as pyrrolizidine alkaloids can be converted through biotransformation and interact with DNA, leading to mutation and cancer (Bode & Dong, 2015).

Lectins, carbohydrate‐binding proteins, are particularly concentrated in legume seeds such as green beans, red kidney beans, and white kidney beans. They have been shown to cause hemagglutination, nausea, and gastroenteritis in humans (Dolan et al., 2010). Lectins are capable of binding to mucosal cells in the intestine, resulting in poor nutrient absorption. It has been reported that the lectins from black beans and soybeans inhibited growth in rats when fed to them at 0.5% and 1% of their diet, respectively. Isolated lectins from kidney beans comprised a lethal dose at 0.5% of the diet in rats after 2 weeks of feeding (Omaye, 2004). Fortunately, some lectins toxicity can be reduced by proper cooking, such as heating (Grant, More, McKenzie, & Pusztai, 1982).

Saponins, natural glycosides, can be found in higher plants. The structure of saponins is a combination of hydrophobic (fat‐soluble), sapogenin, and hydrophilic (water‐soluble) sugar parts (carbohydrate moiety). Saponins play a critical role in plants, including for pathogen protection and as a growth hormone. Health benefits such as anti‐inflammation, immunomodulation, and anticancer have been reported, as well as adverse effects such as hemolysis induction (Podolak, Galanty, & Sobolewska, 2010). Moreover, previous study found that steroidal saponins isolated from *Narthecium ossifragum* cause renal tubular cell (LLC‐PK1) toxicity (Uhlig, Wisloff, & Petersen, 2007).

*Plukenetia volubilis* or Sacha inchi, the Inca peanut, is a tropical plant from the Euphorbiaceae family of the Amazon region (Gillespie, 1993). At present, the seeds have been utilized primarily for oil production. Besides, the boiled or roasted seeds and leaves are also edible (Hamaker et al., 1992). Several chemical composition studies have shown that Inca peanuts contain various health‐promoting compounds such as the phenolic compounds, tocopherol, and phytosterol (Chirinos et al., 2013). The seeds have been assessed for total phenolic content ranging from 64.6 to 80 mg of Gallic acid equivalent/100 g per seed, which is higher than in almonds and macadamias (Kornsteiner, Wagner, & Elmadfa, 2006). β‐sitosterol is the most abundant phytosterol (45.2–50.8 mg/100 g seed) found in the seeds, followed by stigmasterol and campesterol, respectively. However, Inca peanuts are not a good source of carotenoids because they contain a low amount of total carotenoids (0.07–0.09 mg of β‐carotene equivalent/100 g seed). Interestingly, the oil from the cold‐pressed seeds is well known for being healthy because of its high essential fatty acid levels, including ω‐3 and ω‐6, measured at approximately 47–51% and 34–37%, respectively (Fanali et al., 2011). Furthermore, Inca peanut oil has the highest amount of ω‐6 compared to olive, soy, maize, and sunflower oils (Hanssen & Schmitz‐Hübsch, 2011). It has been proven that Inca peanut oil is not only safe but also could increase HDL cholesterol in humans (Gonzales & Gonzales, 2014). Therefore, Inca peanut oil, which is now available in supermarkets as a dietary supplement and as edible oil, demonstrates high economic value. With almost identical tropical weather conditions, Inca peanut has also been imported for growing in Southeast Asia. Besides oil production, seeds can be lightly roasted and served with salt as a snack such as salted peanuts, while the dried leaves can be made into a kind of tea. There is increasing demand in Thailand for the roasted seeds and tea of Inca peanuts, although consumers have expressed various concerns regarding the safety of Inca peanut‐related products.

As there is currently no information regarding the phytotoxin content of Inca peanuts or related products, this study aimed to determine the amount of naturally occurring phytotoxins, including saponins, total alkaloids, and lectins of fresh and roasted Inca peanut seeds and leaves. Cytotoxicity of fresh and roasted Inca peanut seeds and leave extracts in normal cells, including human peripheral blood mononuclear cells, human embryonic kidney cells, human hepatic stellate cells, and mouse fibroblasts, was determined. Moreover, the somatic mutation and recombination test (SMART) in *Drosophila melanogaster* was performed to determine the genotoxicity of the plant samples.

## 2 MATERIALS AND METHODS

### 2.1 Reagents

Saponin, alkaloid, and urethane were purchased from Sigma‐Aldrich^®^ (St. Louis, MO, USA). Thiazolyl blue tetrazolium bromide (MTT dye) was purchased from Panreac AppliChem (Maryland Heights, MO, USA). Lymphocytes Separation Media (Ficoll^™^) was purchased from Capricorn Scientific GmbH (Ebsdorfergrund, Germany). All other chemicals and solvents used were of either analytical or HPLC grade.

### 2.2 Sample and sample preparation

Inca peanut samples, including fresh seeds (FS) and fresh leaves (FL), were provided by Thai Rubber Land and Plantation Co., Ltd. The plantation is located in Chiang Rai Province, Thailand. All samples were shipped to the University laboratory within 1 day after harvesting. Samples were immediately cleaned and air‐dried to prevent mold and undesirable spoilage. Inca peanut tea was prepared as follows: cleaned fresh leaves were dried and cut into small pieces and roasted at 75°C for 1.5 h. Roasted tea leaves (RL) were kept in an aluminum foil bag under vacuum and stored at room temperature. Seeds were roasted (RS) at 120°C for 10 min and kept in the same manner as the roasted tea leaves for further analysis.

### 2.3 Analysis of phytotoxins

#### 2.3.1 Analysis of total alkaloids

The determination of alkaloid compounds that contained nitrogen as part of the ring form was developed by Shamsa, Monsef, Ghamooshi, & Verdian‐riz, 2008. Samples were heated with 30 mL of 100% methanol for 1 h and filtered with filter paper. The samples were evaporated in a rotary evaporator under vacuum at a temperature of 45°C until dried. After, the samples were redissolved by 2 N of HCl and filtered with a filter paper. One milliliter of samples was filled into a conical flask. Subsequently, cleaning was performed using 10 mL of chloroform (in triplicate) and adjusted to neutral pH with 0.1 N sodium hydroxide (NaOH). Five milliliters of phosphate buffer (pH 4.7) and 5 mL of bromocresol green were added. The aliquot was collected in a volumetric flask bottle, adjusted to 10 mL with chloroform, and measured for absorbance at 470 nm.

#### 2.3.2 Analysis of saponins

Saponin analysis was adapted from Wan et al. (2006). Samples (0.5 g) were mixed with 3 mL of 100% methanol, then shaken for 30 s, and sonicated for 2 h at 25°C. The supernatant was collected by centrifugation at 626 g for 5 min. The aliquot was filtered for HPLC analysis. Determination of saponins was carried out using high‐performance liquid chromatography (HPLC) with an Agilent 1100 liquid chromatograph system (Palo Alto, CA, USA). The column used was Phenomenex C18 (4.6 mm × 250 mm, 5 μm). Gradient elution was performed using the mobile phase of (A) water and (B) acetonitrile at the following concentrations: 0–30 min, 18–19% B; 30–35 min, 19–35% B; and 35–60 min, 35–55% B at a flow rate of 1.5 mL/min.

#### 2.3.3 Analysis of lectins

Samples were extracted with 200 mL phosphate buffer saline and sonicated for 15 min. The mixture was centrifuged, and the supernatant was obtained for analysis. Lectin level was determined using a sandwich ELISA Kit (Bovine Lectin‐Galactose Binding‐Soluble 3 (LGALS3)) (MyBioSource Inc., USA). In brief, samples were mixed with 50 μL standard solution, 50 μL controlled solution, and HRP‐conjugated in a 96‐well plate. The mixture was incubated at 37°C for 60 min. After that, the plate was washed four times with a washing solution and 50 μL of chromogen A was added before further incubation at 37°C for 15 min. The reaction was stopped, and absorbance was recorded at 450 nm.

### 2.4 *In vivo* mutagenicity analysis in *Drosophila*

The somatic mutation and recombination test in *Drosophila* (SMART) was employed (Graf, Abraham, Guzman‐Rincon, & Wurgler, 1998; Graf & van Schaik, 1992; Graf et al., 1984). In brief, males of *mwh/mwh* and females of *ORR;flr* ^*3*^ */In(3LR) TM3, ri p* ^*p*^ *sep l(3)89Aa bx* ^*34e*^ *e Bd* ^*S*^ *, Ser* were mated to generate transheterozygous larvae (mwh flr+/mwh TM3). The advantage of the transheterozygous strain is the existence of a biotransformation reaction, which mimics the mammalian biotransformation system (Frolich & Wurgler, 1989). One hundred 3‐day‐old transheterozygous larvae were cultured on standard medium containing water (negative control), 20 mM urethane (positive control), and various concentrations of Inca peanut. The powders of fresh seeds (FS), roasted seeds (RS), and fresh leaves (FL) were substituted with fly food at a ratio of 1:1, 1:2, and 1:4. Thus, the final concentrations of FS, RS, and FL were 145 mg/mL, 73 mg/mL, and 37 mg/mL, respectively. Roasted leaves (RL) were prepared as tea and then substituted with water in fly food for 100%, 50%, and 25%. Thus, the final concentrations of RL were 0.024 mg/mL, 0.012 mg/mL, and 0.006 mg/mL, respectively. The surviving flies were counted within 5 days after the first eclosion and then stored in 70% ethanol. At least 40 wings per treatment were removed and mounted on microscope slides. The phenotypes on the wings, such as single, large, and twin spots, were analyzed under a compound microscope. The data were statistically analyzed as previously reported (Frei & Wurgler, 1988).

### 2.5 Cell viability assay

The cell viability assay was conducted as previously described (Pitchakarn et al., 2017). Human peripheral blood mononuclear cells (PBMCs) were isolated using Ficoll‐hypaque according to the manufacturer's instructions. The mononuclear cells were carefully collected and rinsed twice with ice‐cold PBS pH 7.4 and resuspended in fresh RPMI medium supplemented with 1% penicillin/streptomycin and 10% heat‐inactivated fetal bovine serum. Three cell lines, including embryonic mouse fibroblast (3T3‐L1), human embryonic kidney cell line (HEK293), and human hepatic stellate cell line (LX‐2), were used. All cell lines were cultured in Dulbecco's Modified Eagle Medium (DMEM) supplemented with 1% penicillin/streptomycin and 10% heat‐inactivated fetal bovine serum, and then maintained at 37°C in a 5% CO_2_ humidified atmosphere. At the confluent of about 70–80% in a 96‐well plate, cells were treated with various concentrations of FS, RS (extraction with 80% ethanol) and FL, RL (extraction with hot water, 80°C for 5 min) as previously described (Pitchakarn et al., 2010). After 48 h, the MTT dye was added to the cells and incubated for 4 h at 37°C. Then, the culture medium was removed and DMSO was immediately added to solubilize the formazan dye. The amount of formazan dye, which represents cell viability, was determined using a microplate reader. The assay was performed in triplicate. The 20% and 50% inhibitory concentrations (IC_20_ and IC_50_) were subsequently calculated.

## 3 RESULTS AND DISCUSSION

### 3.1 Phytotoxins in Inca peanut seeds and leaves

This study focused on alkaloids, saponins, and lectins because these compounds are likely to be present in nuts (Dolan et al., 2010; Ganzera, Kruger, & Wink, 2010). Our data showed that fresh and roasted seeds (FS and RS) and fresh and roasted (tea) leaves (FL and RL) contained total alkaloids, saponins, and lectins. However, these phytotoxins were heat‐labile compounds (Table 1). Regarding the different plant parts, FS had the highest amount of total alkaloid content (485 ± 35 mg/kg DW), followed by FL (146 ± 7 mg/kg DW), indicating that the nature of alkaloids in Inca peanuts seems to be accumulated in the seeds rather than the leaves. It has been reported that the glycoalkaloid levels in properly grown potatoes range between 20 and 100 mg/kg and are relatively safe. Based on available human data, the Joint FAO/WHO expert Committee on Food Additives (JECFA) reported that the intake of total glycoalkaloids of 2 to 5 mg/kg of body weight (bw) can cause acute toxicity. A total level of glycoalkaloids more than 3 to 6 mg/kg bw can be lethal (Kuiper‐Goodman & Nawrot, 1992).

**Table 1:** Total alkaloids, saponins, and lectins of Inca peanut fresh and roasted seeds (FS and RS) and fresh and roasted (tea) leaves (FL and RL)

Samples	Total alkaloids (mg/kg DW)	Saponins (mg/kg DW)	Lectins (ng/g DW)
Fresh seeds (FS)	485 ± 35^a^	27 ± 4^b^	0.22 ± 0.03
Roasted seeds (RS)	20 ± 0^c^	5 ± 0^c^	0.15 ± 0.02
Fresh leaves (FL)	146 ± 7^b^	301 ± 14^a^	0.20 ± 0.01
Roasted leaves (RL)	20 ± 4^c^	2 ± 0^c^	0.15 ± 0.06

Values expressed are mean ± standard deviation (SD) of triplicate analysis. The statistical package for social sciences (SPSS, Chicago, IL, USA) 17.0 program was used to calculate for statistical difference by one‐way analysis of variance (ANOVA), with lower letter case indicating a significant difference for each column at *p *<* *.05.

Saponins were detected in both the seeds and leaves of Inca peanuts. The highest amount of saponins was found in FL, approximately 10 times higher than that of FS. Interestingly, comparing between FS and RS, or FL and RL, as seen in Table 1, showed that the amount of saponins was decreased 5 times and 150 times, respectively, after the heating process. The same results were also observed in alkaloid content (Table 1), thus suggesting that the alkaloids and saponins included in the plant samples were heat‐labile compounds. This may be due to the ring structure of saponins being degradable (Uhlig et al., 2007). Other nuts belonging to the Euphorbiaceae family, such as African nut, Barbados nut, and Candlenut, contain saponins ranging from 200 to 500 mg/kg DW (Dolan et al., 2010). Hence, the saponins detected in our study were relatively low and may not contribute to adverse effects.

Lectins are able to induce the cross‐linking of red blood cells. Common foods that contain lectins are wheat germ, beans, peas, spices, and nuts (Vasconcelos & Oliveira, 2004). A type of lectin that is lectin–galactose binding was selected for this study due to a previous study showing that the Areca nut also contains this type of lectin (Dolan et al., 2010). In addition, hemagglutination test was not employed because the presence of agglutinating substances of nonlectin nature may lead to the misinterpretation of the agglutination results. The data showed that all parts of Inca peanuts contained very low lectins, varying from 0.15 to 0.22 ng/kg DW (Table 1). In addition, heat processing seemed to reduce lectin content in processed samples, although it was not statistically significant.

From the experiment (Table 1), it suggests that alkaloids, saponins, and possibly lectins in Inca peanut can be degraded by thermal processing. In support, roasting of *Canavalia plagiosperma* seeds at 120°C for 40 min decreases alkaloids by 2.5 times compared to fresh seeds (Alagbaoso et al., 2016). It is also interesting to study concentration of phytotoxins on degree of maturity and season as they are inter‐related. Nevertheless, the samples used in this study were from a factory which represents the characteristic of commercially available Inca peanut.

### 3.2 *In vivo* mutagenicity analysis in *Drosophila*

To investigate the mutagenic properties of each Inca peanut sample, we used an efficient and versatile *in vivo* short‐term assay involving *Drosophila* wing spot test (SMART) as a tool. It is well documented that the SMART assay has several advantages; (i) the fruit fly has been claimed to be an alternative testing organism, particularly for genotoxicity testing because it is able to detect several types of genotoxins (Batiste‐Alentorn, Xamena, Creus, & Marcos, 1995; Demir, Kocaoglu, & Kaya, 2008; Kaya, Yanikoglu, Creus, & Marcos, 2000), (ii) genotoxicity results obtained from SMART have high levels of reproducibility (Osaba, Aguirre, Alonso, & Graf, 1999), and (iii) SMART is a sensitive, rapid, and inexpensive performance compared to mutagenic study in rodents (Delgado‐Rodríguez, Ortíz‐Martello, Villalobos‐Pietrini, Gómez‐Arroyo, & Graf, 1999). In addition, the transheterozygous strain we used was equipped with high expression of cytochrome P450, thus enabling proper biotransformation similar to mammals (Frolich & Wurgler, 1989; Graf & van Schaik, 1992). The mutant spots on the wing were indicative of several mutagenic events, including point mutation and recombination (Graf et al., 1984, 1998).

**Figure 1:**
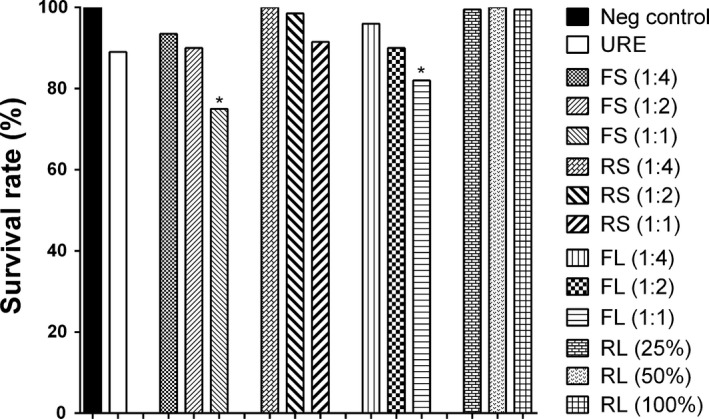
*Drosophila* survival rates after treatment with different concentrations of FS, RS, FL, and RL. Distilled water and 20 mM urethane (URE) were employed as negative and positive control, respectively. The statistical significance was analyzed by Student's unpaired *t* test against negative control. *p <* *.05

To determine survival rates, the third instar larvae of a transheterozygous strain were fed with distilled water (negative control), 20 mM urethane (positive control), and various concentrations of each Inca peanut sample. As shown in Figure 1, all concentrations tested of each sample were not toxic to the tested flies (more than 70% of survived flies) compared to the negative control. Thus, the highest concentration of FS, RS, FL, and RL was used to analyze the mutagenic properties. The mutagenic evaluation of FS, RS, FL, and RL is shown in Table 2. The reference mutagen, urethane, noticeably provoked mutant spots compared to negative control (26‐fold greater than the negative control), which is consistent with previous studies (Budluang et al., 2017; Pitchakarn et al., 2017). Furthermore, the highest concentration of all tested Inca peanut samples did not increase the incidence of total mutant spots compared to urethane after statistical analysis. To avoid any bias, different types of mutant spots, including single, large, and twin spots, were examined separately. The results also exhibited no induction of single, large, or twin spots in FS, RS, FL and RL‐treated group compared to positive control (Table 2). This indicates that the saponins, alkaloids, and lectins detected here are not mutagenic. Regarding the thermal processing of food, it can produce polycyclic aromatic hydrocarbons (PAHs), which have been shown to cause DNA mutations, recombination, and DNA breaks. Indeed, the International Agency for Research on Cancer (IARC, 2010) categorizes PAHs as known, possibly, or probably carcinogenic to humans (Group 1, 2A, or 2B). To this point, after thermal processing of FS and FL, there was no induction of single spots, large spots, twin spots, or total spots compared to urethane‐exposed flies (Table 2), implying that less or no mutagens are formed using our thermal processing method.

**Table 2:** Summary of somatic mutation and recombination test data obtained with the transheterozygous flies after exposure to the highest concentration of Inca peanut fresh and roasted seeds (FS and RS) and fresh and roasted (tea) leaves (FL and RL)

Treatment	Spot per wing (Number of spots from 40 wings)^a^				
Inca Peanut sample	Concentration	Small single (*m* = 2)	Large single (*m* = 5)	Twin spot (*m* = 5)	Total spot (*m* = 2)
Negative control	–	0.48 (19)	0 (0)	0.03 (1)	0.75 (30)
Urethane (URE)	20 mM	17.95 (718)+	5.95 (238)+	2.40 (96)+	26.30 (1052)+
FS (1:1) (1^st^)	145 mg/mL	0.45 (18)−	0.10 (4) −	0 (0)i	0.55 (22) −
FS (1:1) (2^nd^)	145 mg/mL	0.38 (15)i	0.38 (15)i	0.38 (15)i	0.38 (15)i
RS (1:1) (1^st^)	145 mg/mL	0.23 (9) −	0.03 (1)i	0 (0)i	0.25 (10) −
RS (1:1) (2^nd^)	145 mg/mL	0.33 (13) −	0.33 (13) −	0.33 (13) −	0.33 (13) −
FL (1:1) (1^st^)	145 mg/mL	0.78 (31)i	0.03 (1)i	0 (0)i	0.80 (32) −
FL (1:1) (2^nd^)	145 mg/mL	0.40 (16)i	0.40 (16)i	0.40 (16)i	0.40 (16)i
RL (100%) (1^st^)	0.024 mg/mL	0.55 (22)i	0.03 (1) −	0.03 (1)i	0.60 (24) −
RL (100%) (2^nd^)	0.024 mg/mL	0.33 (13)i	0.08 (3)i	0 (0)i	0.40 (16)i

a Statistical diagnoses using estimation of spot frequencies and confidence limits according to Frei and Wurgler (1988) for comparison with deionized water (negative control); + = positive; − = negative; i = inconclusive. Probability levels: α = β = 0.05. One‐sided statistical test “*m*” is an increased mutation frequency compared with the spontaneous frequency (*m* times).

### 3.3 Cytotoxicity test

**Figure 2:**
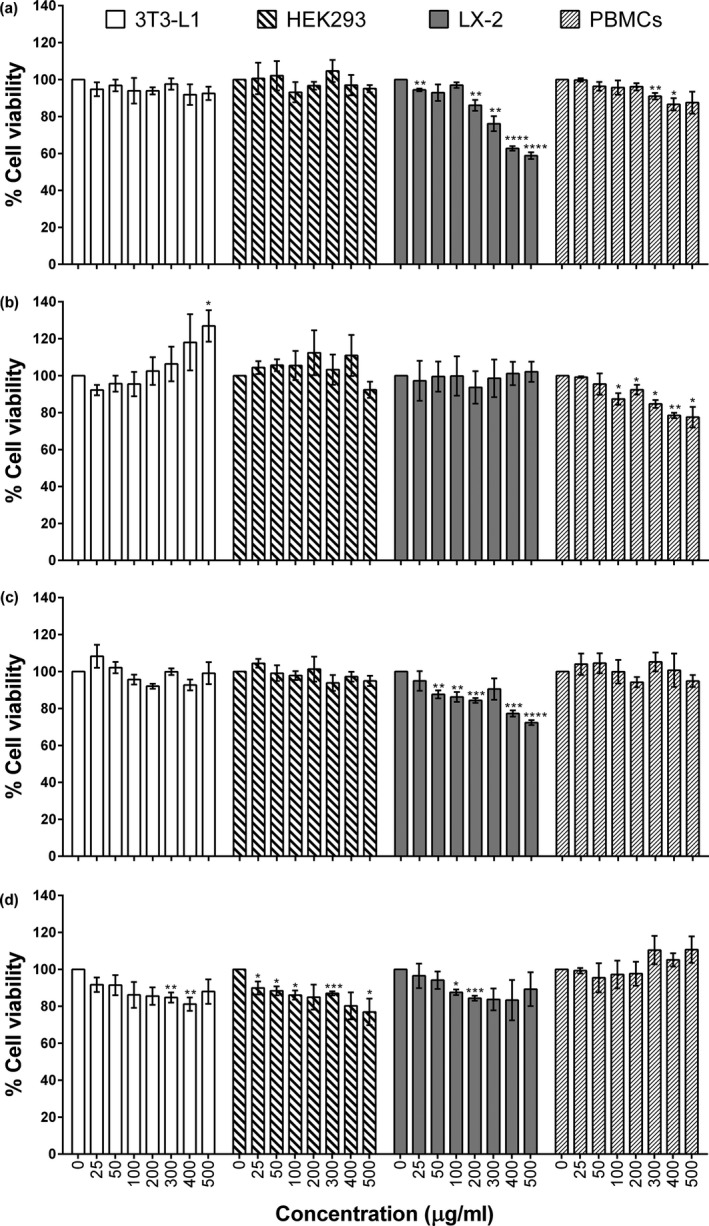
Effect of Inca peanut fresh seeds (FS (a) and roasted seeds (RS (b)) as well as fresh leaves (FL (c) and roasted leaves (RL (d)) on the cell viability of mouse embryonic fibroblast (3T3‐L1), human embryonic kidney cell (HEK293), human hepatic stellate cell line (LX‐2), and human PBMCs**.** Values expressed are mean ± standard error of the mean (SEM) for triplicate analysis. The percentage for cell viability of each cell line (% cell viability) was calculated and compared to the control group. The statistical significance of each cell line was analyzed by Student's unpaired *t* test against its control group. *p <* *.05; **p <* *.01; ***p <* *.001, and ****p <* *.0001

To determine the cellular toxicity of FS, RS, FL, and RL, a variety of cell types that are not derived from cancer cells including embryonic mouse fibroblast (3T3‐L1), human embryonic kidney cells (HEK293), human hepatic stellate cells (LX‐2), and human PBMCs were employed. Cells were exposed to various concentrations of each extract ranging from 25 to 500 μg/mL for 48 h. After that, the IC_20_ and IC_50_ were determined. As shown in Figure 2a, FS extract, compared to vehicle control, displayed no significant differences in any tested cells, albeit mild cytotoxicity was specifically observed in hepatic stellate cells (LX‐2) with IC_20_ at 250 μg/mL and IC_50_ at 500 μg/mL (Table 3). In contrast to FS extract, RS extract was safe (IC_50_ ˃ 500 μg/mL) in all tested cell types, including LX‐2, implying that heat processing of Inca peanut seeds may inactivate or destroy mild hepatotoxins. Figure 2c and d represent the cytotoxicity of FL and RL extract. The obtained data showed that FL exhibited mild hepatotoxicity to LX‐2 cells, as found in FS (IC_20_ at 400 μg/mL Table 3). Further, the severity of LX‐2 toxicity seems to depend on the concentration of alkaloids (Table 1). Alkaloids have been reported for their ability to irritate the gastrointestinal tract. The heat‐labile properties of alkaloids have also been revealed (Alagbaoso et al., 2016). Thus, it is tempting to speculate that the mild toxicity observed in LX‐2 after FS exposure may be due to the presence of alkaloids. Furthermore, the presence of a biotransformation system in hepatic cells may sensitize them to alkaloid toxicity. Together with the fact that alkaloids are heat‐labile, no cytotoxicity was observed after exposure to roasted leaves (RL) (Figure 2d). Interestingly, Nascimento et al. (2013) found that the ethanolic fraction from Inca peanut fresh leaves (FL) did not cause cytotoxicity in 3T3‐L1 cells (Figure 2c), but did inhibit cell growth in two cancer cell lines (Hela and A549 cells), consistent with our data. This indicates that the FL extract may possess anticancer properties and is harmless to normal cells, which are expected of chemopreventive agents.

**Table 3:** Summary of 20% and 50% inhibitory concentrations (IC_20_ and IC_50_) of Inca peanut fresh and roasted seeds (FS and RS) and fresh and roasted (tea) leaves (FL and RL) on mouse embryonic fibroblast (3T3‐L1), human embryonic kidney cell (HEK293), human hepatic stellate cell line (LX‐2), and human PBMCs

Sample	Cell line	IC_20_ (μg/mL)	IC_50_ (μg/mL)
FS	3T3‐L1	˃500	˃500
FS	HEK293	˃500	˃500
FS	LX‐2	250	500
FS	PBMCs	˃500	˃500
RS	3T3‐L1	˃500	˃500
RS	HEK293	˃500	˃500
RS	LX‐2	˃500	˃500
RS	PBMCs	˃500	˃500
FL	3T3‐L1	˃500	˃500
FL	HEK293	˃500	˃500
FL	LX‐2	400	˃500
FL	PBMCs	˃500	˃500
RL	3T3‐L1	˃500	˃500
RL	HEK293	400	˃500
RL	LX‐2	˃500	˃500
RL	PBMCs	˃500	˃500

## 4 CONCLUSION

Fresh Inca peanut seeds (FS) and leaves (FL) contain various levels of heat‐labile phytotoxins, including alkaloids, saponins, and lectins. High and chronic consumption of FS and FL should be of concern in order to reduce hazard risks. Roasting can most effectively reduce these phytotoxins, and hence, thermal processing should be applied before the consumption of Inca peanut seeds and leaves. Further studies including acute and chronic toxicity in animals may provide additional clarity for determining a safe intake level of Inca peanut seeds and leaves.

## CONFLICT OF INTEREST

WS and PT received a grant from Thai Rubber Land and Plantation Co., Ltd., Chiang Rai, Thailand. The funder had no role in study design, data collection and interpretation of the data, decision to publish, and manuscript preparation. All authors declare that there are no conflict of interests.

## ETHICAL REVIEW

The *Drosophila* study was approved by Mahidol University‐Institute Animal Care and Use Committee (MU‐IACUC) (COA.No.MU‐IACUC 2017/025).
